# Exploring rehabilitation potential in older people living with frailty: a qualitative focus group study

**DOI:** 10.1186/s12877-021-02107-y

**Published:** 2021-03-06

**Authors:** A. Cowley, S. E. Goldberg, A. L. Gordon, M. Kerr, P. Logan

**Affiliations:** 1 Institute of Care Excellence, Nottingham University Hospitals NHS Trust, Derwent House, City Campus, Hucknall Road, Nottingham, NG5 1PB UK; 2 School of Health Sciences, University of Nottingham, Nottingham, UK; 3 School of Medicine, University of Nottingham, Nottingham, UK; 4 University Hospitals of Derby and Burton NHS Foundation Trust, Derby, UK; 5 NIHR Applied Research Collaboration East Midlands (ARC-EM), Nottingham, UK; 6 Nottingham CityCare Partnership CIC, Nottingham, UK

**Keywords:** Rehabilitation, Frail elderly, Geriatric assessment, Decision-making

## Abstract

**Background:**

Rehabilitation interventions are frequently cited as key in supporting frail older people’s recovery following periods of decompensation and acute ill-health. Clinicians are required to make decisions about a patient’s potential to respond to rehabilitation. ‘Rehabilitation potential’ decisions can determine access to services. In acute settings clinicians have limited time to assess and work with patients, families and carers. The complexities of ageing, recovery, rehabilitation and frailty may not be fully appreciated. This study aimed to explore multiple perspectives of the concept of rehabilitation potential and how it is assessed in older people living with frailty in the acute healthcare setting.

**Methods:**

Five focus groups with a purposive sample of 28 participants which included clinicians and members of the public were conducted. Analysis comprised a thematic approach using the Framework method.

**Results:**

Rehabilitation potential was found to encapsulate a complex decision-making process where clinicians judged an individual’s ability to benefit from and participate in targeted rehabilitation. They asked, “Will it work?”, “Is it wanted?” and “Is it available?” In order to predict who would benefit from rehabilitation interventions, clinicians assessed a range of holistic clinical and non-clinical factors. An iterative approach to assessment delivered by a multi-disciplinary team, centred around patient and carer needs and wants was needed to accommodate complexity. Participants believed that everyone had some form of potential but this was dependent on availability of rehabilitation resources and conceptualisations of frailty and rehabilitation. Tensions between iterative approaches to rehabilitation potential assessment and the realities of rapid decision making in the acute hospital setting were found.

**Conclusion:**

Rehabilitation potential decisions involve a complex process of multidisciplinary decision-making and prognostication on the likely outcome and benefit from rehabilitation programmes. These findings lay the foundation for developing structured approaches to rehabilitation potential decision making tools and guidance.

## Background

Frailty is a concept used by healthcare practitioners and is currently understood as a state of increased vulnerability to poor resolution of homeostasis after a stressor event [1]. It is associated with an increased risk of disability, delirium, hospitalisation, pain, falls and death, placing individuals at higher risk of adverse outcomes [2–4] and dependence in activities of daily living. Rehabilitation interventions are key in supporting recovery following periods of decompensation and ill-health [5]. Geriatric rehabilitation has been defined as a “*multi-dimensional approach of diagnostic and therapeutic interventions, the purpose of which is to optimise functional capacity, promote activity and preserve functional reserve and participation*” [6]. Rehabilitation adopts a holistic approach, enabling individuals to maximise their well-being, activities of daily living, function and social integration [7]. It seeks to restore personal autonomy to those aspects of daily life considered most relevant by service users and their family or carers [5]. Rehabilitation delivery is a process as opposed to a one-off event [8]. Clinicians, in partnership with patients and carers seek to obtain a broader understanding of the nature and causes of underlying conditions, and mitigate the impact that these have on individuals through rehabilitation programmes [9]. However, clinicians are required to make nuanced, complex and dichotomous decisions about patients’ rehabilitation requirements in the face of growing complexity of service provision, patient needs and expectations. In the acute hospital setting, they have to make rapid and complex decisions, about who will respond to rehabilitation. Clinicians increasingly recognise that positive gains in terms of quality of life, function and social participation can be achieved with even the most physically and cognitively impaired adults [10]. The clinical currency in which these decisions are transacted is through the use of the term rehabilitation potential.

Rehabilitation potential in relation to older people was first conceptualised in the 1950s [11–13]. Reynolds et al. [12] proposed that rehabilitation potential was rated by consultant physicians as either ‘definite’, ‘slight’ or ‘none’ in relation to the anticipated benefit of rehabilitation services to restore function. It has been used by researchers as a retrospective label to describe how well a patient improves functionally in response to rehabilitation [14, 15] – “the patient had rehabilitation potential”. Researchers have also used it to define a prognostic concept of how rehabilitation might restore activities of daily living [16, 17], comprising considerations around a patient’s psychological ability to take part in rehabilitation [18], and the impact of the rehabilitation environment on the chances of recovery [19, 20]. Cameron and Kurle [21] suggested that frailty was a significant mediating factor in establishing the rehabilitation potential of older people living with frailty, but did not describe what was meant by rehabilitation potential, nor how clincians understood or assessed this in the frail older population.

In clinical contexts, decisions about rehabilitation potential can determine what rehabilitation services a patient can access [10, 22]. Frailty can modify how older people respond to rehabilitation [21]. Older people with frailty are not always able to regain function once it is lost and assessments of rehabilitation must include restorative and adaptive rehabilitation approaches which combine to help support activities and participation [23, 24].

There is no universally agreed, systematically assessed or explicitly operationalised model or clinical guidelines that help clinicians to make consistent, transparent, patient-centred and evidence-based decisions about rehabilitation potential. Understanding how rehabilitation potential is: assessed, operationalised, and which factors influence decision-making and patient and carer involvement in the process are essential for holistic and evidence-based approaches to frailty rehabilitation. Consequently there is a need to develop a more robust and explicit framework for assessing and operationalising rehabilitation potential. For these reasons, we conducted a qualitative focus group study which aimed to: (i) explore how clinicians, patients, carers and academics understood rehabilitation potential in older people living with frailty; (ii) explore how rehabilitation potential was assessed in the hospital and community setting and; (iii) to identify evidence to inform the development of a tool to support consistent decisions about rehabilitation potential.

## Methods

Qualitative inquiry is ideally suited to capture the complexity of rehabilitation potential and can provide direct access to what people do in real life rather than asking them to merely comment on it [25]. Focus groups promote group discussion through open ended questions and can lead to a rich understanding of how people make sense of their social worlds and the meaning of events [26]. Rather than producing individual accounts, focus groups allow for socially shared tacit knowledge to be generated, maintained and changed through dynamic group discussions [25].

We held five focus groups across the East Midlands Region in the United Kingdom between February and March 2018. By carrying out focus groups across the region it was possible to explore experiences outside one system of local working and practice, enhancing the transferability of the findings. We aimed to recruit a heterogeneous group of participants who were typically involved in or received assessments of rehabilitation potential and rehabilitation programmes. This included clinicians, patients, carers and academics. Although this had the potential to create issues with social power arising from professional hierarchies, participants were chosen from these groups to generate new insights into rehabilitation potential and stimulate group discussion. Social power refers to the potential or ability to influence others in a group setting [27]. A number of actions were taken to mitigate against this. Group discussion rules were developed and shared at the start of each group. These highlighted the need to keep discussions held in the group confidential and asked participants to respect others experiences and opinions. These measures sought to foster an environment where participants felt comfortable to share potentially sensitive issues and freely express their opinions without consequence or distress. Participants were offered support from the Patient Advice and Liaison Service and the NHS Employee Assistance helpline to discuss any sensitive, challenging or difficult subjects that arose. There were no instances where this occurred.

**Fig. 1:**
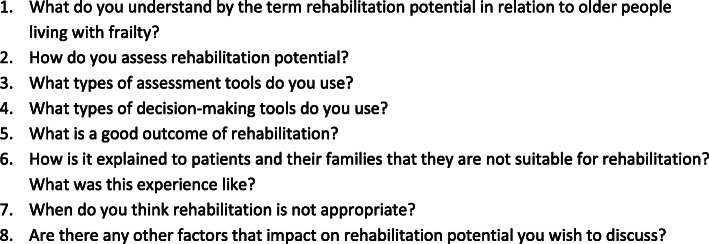
Focus group topic guide

We developed the focus group topic guide (Fig. 1) using findings of a systematic mapping review [28], supplemented with the clinical experiences of the study team, including our Patient and Public Involvement (PPI) member (MK). We piloted the schedule with local clinicians and members of a Dementia and Frail Older people PPI group. It was designed to support flexible and organic discussions on rehabilitation potential and to provide structure to ensure objectives were met in a timely manner.

We held focus groups in venues away from healthcare facilities and at dates and geographical locations convenient to participants. They lasted between one and two hours. The groups were facilitated by AC, a female clinical academic physiotherapist and by MK, our PPI team member and lay researcher, who observed group dynamics, verbal and non-verbal communication with a specific focus on empowering non-professional members of the group. Data were digitally audio-recorded and transcribed verbatim.

### Participants

Clinical and academic professional bodies, and patient and public involvement groups advertised the study to healthcare professionals, patients and carers through study posters. Interested participants were invited to contact the researchers directly and were screened by phone or email against the following inclusion criteria: professional participants with experience of assessing rehabilitation potential in older people living with frailty in the acute hospital setting or delivering rehabilitation to older people in the acute, sub-acute or community setting: patients or carers with experience of receiving a rehabilitation assessment or period of rehabilitation, caring for someone previously in receipt of rehabilitation or being told they or their relative had no rehabilitation potential. A participant information sheet was sent and they were invited to a focus group. We obtained written informed written consent prior to data collection. Mental capacity was assumed to be present unless proven otherwise in line with the Mental Capacity Act [29]. Participants who did not feel able to participate in a group setting due to difficulties with communication, cognition, specific concerns or experiences with participating clinicians were offered one-to-one interviews to ensure that a range of opinions were obtained. There were no instances of this occurring.

### Sampling

We used purposive sampling to obtain a variety of experiences, professions and views. Sampling and data collection ran concurrently. Sample sizes in qualitative research are frequently driven by the concept of data saturation, defined as “*the point in data collection and analysis where new information produces little or no change to the codebook”* [30]. Guest et al. [31] found that 80% of analytical themes were found within two or three focus groups and 90% within three to six groups. This suggests that a sample size of three to six groups led to the identification of the majority of themes. Focus groups typically have between six and ten participants [26]. Ten participants were recruited into each group with the aim of achieving an overall sample size of 24.

### Data analysis

Data were thematically analysed using the Framework Analysis [32] approach. Framework analysis has been suggested as useful in the development and evaluation of complex interventions. It allows both inductive (emergent) and deductive (a priori) coding and provides a structured, robust and transparent method for data analysis [32–34]. A deductive framework was constructed to take into account the study aims, findings from a prior mapping review [28] and theories of intervention design [35]. This included definitions of rehabilitation potential, how, where and when it was assessed and factors which influenced decision-making. The framework was reviewed by the study team to ensure that resulting codes or labels were grounded in and supported by the data. Repeated listens to the audio-recordings of interviews were conducted alongside a review of field notes. Audio-recordings were then transcribed. Initial analytical notes, key ideas and impressions were generated. Two researchers (AC and MK) read each line of the first two transcripts applying codes independently. AC used NVivo 11 and MK completed this manually. MK, a PPI researcher, received specialist training and support in qualitative data analysis, described elsewhere [36]. Data supporting a priori codes from the pre-defined framework were identified, along with emergent themes from the transcripts. AC and MK kept reflective diaries and these were integrated alongside initial codes to increase analytical insight and credibility of the findings.

AC and MK then met to review and compare codes. These were then arranged into established and emerging themes. Themes describe patterns within the data, capturing items of importance associated with the research question and aims [37]. The framework was reviewed by the wider study team to improve rigour [33]. This process was conducted iteratively in parallel with further focus groups. AC and MK analysed the remaining transcripts, developing new codes and integrating these into the analytical framework until no new codes or themes emerged. To understand how themes were replicated, or differed between transcripts, data were charted onto a matrix where each participant’s responses were listed under the themes and supported with relevant verbatim excerpts. Quotes were presented from individual participants and group discussions which illustrated the essence of each theme. This process allowed for data to be explored across cases and themes [33] and for the identification of disagreements and deviant cases. Deviant cases involve identifying evidence which does not support original theories or disagreements between participants and inclusion increases the credibility of findings and mitigates against the tendency to select data which supports researchers’ assumptions [25].

### Ethical approval

The study was reviewed by the Yorkshire & The Humber – Bradford Leeds Research Ethics Committee on 21st November 2017 and a favourable opinion was given on 3rd January 2018 (17/YH/0356 IRAS project ID 227288). The study was conducted according to the Declaration of Helsinki. Written informed consent was obtained from all participants prior to participation and they were all assured that they could withdraw their consent at any time without consequences.

## Results

Twenty-eight participants took part in five focus groups, which ranged in size from three to eight participants (Table 1). Professional participants worked in a variety of settings including: specialist geriatric rehabilitation units, healthcare of the older people’s wards, community rehabilitation services and dementia services. The groups were predominantly recruited from UK based healthcare professionals but a visit from some Dutch Elderly Care Physicians to the unit of one group of study participants, allowed their opportunistic recruitment. Elderly care physicians are primary care doctors with specialist training in the care of older people who select patients for rehabilitation and support them through rehabilitation programmes in the Netherlands [38].

**Table 1:** Participant information

Profession	Group 1	Group 2	Group 3	Group 4	Group 5	Total
**Physiotherapist**	3	1	1	2	0	7
**Occupational therapist**	2	1	2	1	0	6
**Rehabilitation service users/carers**	0	2	0	1	2	5
**Elderly Care Physician**	0	0	0	4	0	4
**Geriatrician**	0	2	1	0	0	3
**Nurse**	0	0	0	0	1	1
**Commissioner**	0	1	0	0	0	1
**Social worker**	0	1	0	0	0	1
**Total**	5	8	4	8	3	28

We found that rehabilitation potential was understood by asking: “will it work?”, “is it wanted?” and, “is it available?” These three themes will be presented and discussed.

### Will it work?

Central to the concept of rehabilitation potential was the notion of ‘will it work’. This theme described how participants made predictions about which patients would benefit from rehabilitation and how benefit was understood and measured. Rehabilitation potential was found to involve a complex process of prognostication and clinical judgement of which individuals would benefit from rehabilitation. Some participants held the belief that everyone had some form of potential, which was highly individualised and influenced by patients’ functional needs and wants. Therefore a unifying definition of rehabilitation potential was deemed to be less clinically useful.

*“The patient may have been only furniture walking around the house before … so how can we measure all those things to determine what their potential is?* [Occupational therapist five]

*What the potential is, is determined by what activities they [patients] need to do. My potential is different from your potential. So I think it is person determined, activity determined.”* [Physiotherapist five]

Focus group three

Participants’ discussions around measuring rehabilitation benefit and potential lead to interesting and discordant insights into understanding rehabilitation outcomes. Some participants held the view that rehabilitation should always seek improvement with measurable gains in function and independence. Given the fine balance between dependence and independence, small gains were seen to be significant in supporting independent living and quality of life.

*“Patients who have an acute event, hip fracture, stroke, they have potential to gain something but there are other patients that have chronic disease like heart failure, they have a sloped line.”* [Elderly Care Physician one, group four]

However, other participants expressed the view that rehabilitation that sought to maintain, slow or manage the decline of abilities should be of equal value.

*“In terms of people's potential, I think it is about looking at how big that potential needs to be. So for some people it could be very small steps and it is not necessarily about them being able to do things for themselves that they can't do … can they adapt the way they do things, can they compensate and find different ways of doing things, to be able to have some purpose.* [Occupational therapist four]

*I think that is a really good point … if they’ve got a life limiting illness, for example end stage heart failure, they don’t have the reserve to do lots and lots of rehab. Maybe all they need to be able to do is sit in a chair, have tea and watch TV.* [Geriatrician  three]

*Yes that is rehabilitation; it is helping someone to manage their energy and to adapt to their environment.”* [Occupational therapist four]

Focus group three

Participants spoke of the challenges they faced in working out if change or improvement was feasible. They reported the need to understand underlying trajectories of diagnoses, disease progression and rehabilitation response.

*“How do we distinguish between general decline and when somebody comes out of hospital what their potential is? Because obviously, you've got comorbidities that have an impact on function, it is a fine balance between what they also perceive as their potential. I often try and pin it down a bit more when I am taking their initial histories to okay, literally in the weeks following, the weeks prior to coming into hospital and you're feeling well what were you able to do?”* [Occupational therapist five, group three]

Iterative approaches enabled the multi-disciplinary team to understand how benefit was measured or gains “carried-over” between assessments. This was said to provide invaluable information on the perceived success of rehabilitation programmes.

*“You need carryover … it’s something you need to happen where we [patient and clinician] agree to a goal and work towards that goal.* [Physiotherapist seven]

*Rehabilitation is not a half hour session on one day, rehabilitation takes days, weeks.* [Physiotherapist six]

*Exactly, it’s iterative*.” [Elderly Care Physician two]

Focus group four

Dissonance was identified between professional and carer participants. Some believed only healthcare professionals could predict benefit, having greater experience of multi-morbidity, rehabilitation response and evidence in comparison to patients and their families. Patient and carer participants vehemently disagreed with this stating that they could understand what was achievable and realistic and hence influence understandings of rehabilitation potential, if adequate support and education were given; thereby empowering them to be equal partners in rehabilitation potential assessments and rehabilitation intervention delivery.

*“I just want to be educated … . I wanted to speak to someone that could tell me how I could achieve optimal health and well-being. I knew I couldn't be cured because two consultants had told me of the issues*. [Member of public two]

*It’s the explanation. I was thinking about somebody who wanted to walk but you can’t walk until you’ve sat up. And trying to get that message across, that actually sitting on the side of the bed is the first goal before standing up.”* [Occupational therapist three]

Focus group two

### Is it wanted?

This theme considered the role of volition and motivation in establishing individuals’ rehabilitation potential. Rehabilitation was viewed as an active partnership which required a willingness to participate in order to gain benefit and desired outcomes. An understanding of an individual’s rehabilitation potential was said to emerge over multiple assessments and through trials of rehabilitation. Participants stated that an iterative approach allowed for complexity and fluctuations in function, which are common in frailty and dementia, to be understood, so that potentially life-changing decisions were not made at a single time point. This was particularly important during the acute phase when individuals were still undergoing medical treatment where assessments during this time were felt to produce an unfair representation of abilities.

“*They've had this acute event and they have been in bed for a week because they have had really bad pneumonia or whatever and they are already teetering on the edge of not doing much for themselves because they are used other people doing it for them … we haven’t given them a chance and that is constricted by the limitations of the time that you have.”* [Geriatrician three, group three]

This approach was said to be challenging in operationalise in the presence of cognitive impairment and communication difficulties.

*“There have been some situations when people have said that this particular individual may not be capable of rehabilitation or is at a stage of dementia that actually trying to rehabilitate them would not be appropriate because of lack of capacity, lack of ability and the disease itself actually impacting on that level of rehabilitation.* [Nurse one]

*I am quite sure that this would apply to my mother-in-law who has Alzheimer’s and her brain has shrunken. She is frail and 90 and suffers from hallucinations and delirium. I would certainly say, but I’m still not sure why some effort could not be made to offer something that might enable her to function better. [Member of public four]*

*I agree with you … as a clinician, if someone has dementia, sometimes it depends on the level of dementia, it is difficult for people with cognitive impairment to take this on board.” [Nurse one]*

Focus group five

Family members reported experiencing challenges when the notion of rehabilitation potential was linked to motivation or their relative’s involvement in rehabilitation assessments or programmes. This was found to be particularly pronounced in the presence of Alzheimer’s and other dementias when assessments were completed in the ‘artificial’ hospital environment.

*“[Referring to her mother] the hospital was adamant that they weren’t going to let her out until she could walk. We were told she wasn’t very compliant. She was adamant she wouldn’t walk in hospital and we tried very hard to tell the hospital that she would walk if she got out. They sent her home; she got out the ambulance and walked in.”* [Member of public five, group five]

Individualised assessments which considered individual patient needs, wants and abilities were believed to enhance motivation and participation. Some participants preferred such approaches over standardised assessments.

*“I don’t try to assess someone on something that they don’t do regularly before. So, if they don’t drink hot drinks, I wouldn’t drag them to the kitchen.* [Occupational therapist one]

*It’s about the context of it isn’t it because in hospital you are in the routine of the ward, it doesn’t fit with what maybe they are doing at home so they might not engage. [Occupational therapist two]*

*That’s one nice thing about working in the community.” [Physiotherapist three]*

Focus group one

This view was echoed by carer participants who placed a high value on individualised and person-centred approaches to rehabilitation assessments but often reported that these were not experienced in the acute hospital setting.

*“It isn’t individualised and so things can drop through … .because there was the tick box system of can she do this can she do that … a couple of questions to me of what is most important to you and I would have said my eyesight and then that should have set off actions to help me.”* [Member of public three, group four]

Family involvement and support were seen as crucial in determining rehabilitation potential, particularly where patients lacked capacity and insight. Individuals with support from family or friends were viewed as more likely to make sustained gains in rehabilitation and continue with the intervention outside of allocated therapy time.

*“Involve the family if it is appropriate, particularly if the person doesn't have the capacity, then the family become more important … you try and build up trust and build a relationship more so than you would normally.”* [Nurse one, group five]

*“I would have appreciated being given advice to enable me to do more for myself … they should have listened to me when I said I was allergic to that medication but they didn’t.”* [Member of public two, group two]

### Is it available?

Availability of rehabilitation resources was found to be a critical component of rehabilitation potential. This theme described the challenges participants faced in accessing the type, intensity and availability of rehabilitation services available to patients and the ethical dilemmas in working with limited resources. Participants spoke of their belief that everyone should be given fair access to rehabilitation, regardless of age and diagnosis but that they faced dilemmas when working in systems where resources were limited. Some participants reported instances where they had not referred individuals whom they deemed unlikely to make significant gains to or participate in rehabilitation programmes but that they felt ambivalent about this.

*“Some of it is about what we are prepared to invest in. So if we say everybody can make some improvement in their life, how much energy are we as a service going to put into the person or are we only bothered if it’s going to save some other resources for health or social care in the short term or preventatively in the long-term. Or do we see some value for that person … I find it difficult as a therapist because no matter how disabled you are at the time; you can probably improve.* [Occupational therapist four]

*I am with you on that. But I think it goes round to be more business orientated than what we were initially established to be … I think we are losing that if the patient is a number … they should be classed as a person, looking at all aspects of that persons’ life.”* [Physiotherapist five]

*It’s difficult though isn’t it? There aren’t infinite resources and I do believe, even as an OT that we’ve got to use resources wisely. How do you determine who’s got potential, where is the money going to be best spent, where are the efforts going to be best used?”* [Occupational therapist five]

Focus group three

This view was echoed by members of the public who judged that everyone should have fair access to rehabilitation and that some kind of benefit was, in most cases, achievable. However, rehabilitation that met individuals’ needs was not always readily available or provided at optimal intensity. Professional participants reported that this placed them under a great deal of pressure and led to ethical dilemmas when allocating rehabilitation in resource-limited systems which had fixed and frequently outdated notions and models of rehabilitation.

*“It’s a lot of responsibility to make those decisions, especially with closed decisions between care home or last attempt for home.”* [Occupational therapist one, group one]

This view was expanded further through discussion with a commissioner who discussed how money was allocated within a pressured system.

*“Is there a need for this service? Is there an evidence-base and how much does it cost? We have to weigh up with the pot of money we’ve got for the system and it goes on those things that are proven to work, where we know we are going to get the best bangs for our buck.”* [Commissioner one, group two]

One geriatrician proposed that greater transparency was required in the decision-making process and how this was communicated with other healthcare professionals, patients, families and their carers.

*“Is it available? Yes, we are in a rationed system, but we should at least be open and honest and transparent. So, if we are saying we aren’t going to do it because it’s not available, let’s not say we’re not going to do it because the person can’t possibly benefit from it.” [Geriatrician one, group two]*

In some cases, this led to the patients being prematurely labelled as having no rehabilitation potential due to narrow understandings of what rehabilitation could achieve and a lack of in-depth assessments in the acute setting.

## Discussion

This study found that the term rehabilitation potential was poorly defined in clinical practice and meant different things to different participants at different times. It involved predicting who would benefit from and participate with rehabilitation programmes and was driven by three main questions:” will it work?”, “is it wanted?” and “is it available?” Assessments of rehabilitation potential were found to be understood through holistic assessments completed over multiple time points by an experienced multi-disciplinary team with a focus on what was important to the patient.

Assessments of rehabilitation potential have frequently taken a binary approach – patients either have or don’t have rehabilitation potential [14, 15, 19, 39]. This study suggests a more nuanced approach is required, taking account of the fluctuations and complexities that go hand in hand with frailty and the acute care setting. Decisions may therefore be more tentative and less dichotomous. The way in which participants sought to gain a comprehensive picture of patients’ underlying comorbidities and active medical conditions as part of broader assessments, share some commonality with Comprehensive Geriatric Assessment models of care [40]. Geriatric rehabilitation and Comprehensive Geriatric Assessment Models of care embrace an iterative approach to diagnosis, prognostication and rehabilitation interventions. This study found the concept of rehabilitation potential shared the principles of these approaches, but in the acute hospital setting this was often compromised by the need to make rapid and single-time point decisions.

Predicting recovery in older people living with frailty, and rehabilitation potential following a period of acute ill health, was found to be challenging. Cameron and Kurle [21] suggested that such questions depend on whether frailty is conceptualised as a temporary or permanent state, whereby rehabilitation will be seen as likely to lead to improved abilities if conceptualised as temporary. However, a major problem with this conceptualisation of frailty is that whilst the physical decline associated with an acute event may be reversible, the reversibility of the ‘frailty’ itself is less certain. Clinicians need to be cognisant of these distinctions to address them in a sufficiently nuanced way. These contrasting concepts of frailty were identified in participants’ responses where they wrestled with understanding what could be improved with rehabilitation, or when adaptive and palliative approaches to rehabilitation were required. Whilst approaches that seek improvement in function and quality of life have predominated geriatric literature [41], there are suggestions that palliative approaches to rehabilitation could help address the clinical implications of these differing approaches [23, 24] and clinicians understanding of rehabilitation potential. Further research is required to understand the role that rehabilitation may play in reversing frailty.

This study found that motivation was a critical component of rehabilitation potential assessments. Motivation for rehabilitation influences participation and success in terms of physical function [23]. It has previously been linked to rehabilitation potential [17, 19]. Exploring an individual’s motivation, emotions and goals allows for an understanding of how they will react with rehabilitation programmes [42], whereas prognosis or prediction considers variables and outcomes.

Trials of rehabilitation which assessed patient participation, motivation and response were found to inform decisions on rehabilitation potential. This was found to be challenging amongst those with permanent or temporary cognitive impairment. In relation to people living with dementia, low rehabilitation potential and motivation have been suggested as significant factors for poor rehabilitation response and rehabilitation outcomes [43]. Goodwin and Allan [10] have proposed that such assumptions are not based on evidence, rather they are based on clinicians’ preconceived expectations of what individuals with dementia are capable of achieving. In acute hospital settings, professional participants spoke of the pressures they faced in making rapid decisions about rehabilitation potential, and the tensions between individual versus system level needs. This often led to decisions being made ‘for’ the patient rather than ‘with’ the patient, in direct conflict with modern person-centred approaches to rehabilitation and frailty management [44].

This study suggests that clinicians also take into account the availability of rehabilitation resources when making decisions about rehabilitation potential in frail older people. These findings are consistent with other studies into rehabilitation potential in stroke, Acquired Brain Injury [45, 46] and geriatric rehabilitation [19]. Clinicians can find the task of identifying rehabilitation potential challenging in an environment where resource availability can interfere with clinical judgements [22]. This is an important consideration, as individual patients may fail to reach their potential as a consequence of resource limitations, even where other factors meant that they might have responded well to rehabilitation.

Participants in this study spoke of the ethical dilemmas they encountered in navigating decision-making in frailty rehabilitation. This study found that participants respected decision-making capabilities of autonomous individuals but spoke of the challenges they faced when dealing with individuals with either permanent or temporary cognitive impairment. The importance of justice also featured strongly. Participants wrestled with the desire for equality of access to rehabilitation for their patients, regardless of age, cognition, degree of frailty or burden of comorbidity, but this was tempered by the knowledge that resources were limited and had to be rationed where need and benefit were perceived to be the greatest [47]. Age-based rationing of services presents practical and ethical challenges in terms of prioritisation and allocation of services for service users, providers and commissioners.

### Strengths and limitations

Strengths of this study include the diversity of participants recruited to the focus groups, which increased the generalisability and transferability of the findings through wider insights into rehabilitation potential in different healthcare systems. However, the sample was biased towards professional participants which may have affected the strength of patient and carer representation in the data. Including the voice of patients and families, ensured that their important perspectives were included but we were unable to recruit any participants with cognitive or communication impairment. Transferability of findings could have been enhanced further by identifying professional participants years in practice and time working with geriatric patients.

Skilled facilitation is an essential prerequisite for successful focus groups [48] and can affect the validity and reliability of findings. A specialist focus group and PPI facilitator observed preliminary groups, providing feedback on the style and effectiveness of AC and MK. AC’s personal experience and profession may have influenced the thematic analysis and introduced bias into the findings. Participants’ responses and involvement in group discussions may have been influenced by existing relationships with other participants and the study team. Participants may have been reluctant to express beliefs that were contradictory to other group members. These risks were mitigated by the process of reflexivity, which sought to integrate these insights into the analytical framework and by the inclusion of MK as a PPI co-investigator. MK collected and analysed data thus mitigating against one perspective dominating analysis [34]. As a consequence, greater emphasis was placed on patient and carer experiences and the need to foster family involvement in assessments of rehabilitation potential.

The opportunistic recruitment of Elderly Care Physicians from the Netherlands, whilst working in different models of healthcare, revealed that rehabilitation potential was a concept not isolated to the UK and so the inclusion of Dutch participants increased the generalisability and transferability of the findings through wider insights into rehabilitation potential in different healthcare systems. Additionally, it provided the opportunity for deviant accounts and constructs of rehabilitation potential to be explored.

## Conclusions

This study suggests that rehabilitation potential encapsulates complex decision-making processes where clinicians, in partnership with patients, make clinical judgements on an individual’s ability to benefit from and participate in targeted rehabilitation programmes. Participants believed that everyone had some form of rehabilitation potential, but this was dependant on their conceptual understanding of rehabilitation and the availability of current rehabilitation interventions to meet individual needs. There was a sense that rehabilitation of sufficient intensity, duration and specialisation was not available to meet all patients’ needs. Ethical dilemma’s associated with resource availability and allocation featured strongly in participants’ responses and the challenges they faced in navigating these in the hospital setting and rationing of resources.

This study provides a structure for rehabilitation potential decisions - “Will it work?”, “Is it wanted?” and “Is it available?” –and lays the foundation for guidelines or assessment tools which make such decisions more transparent and consistent. Future research should focus on the development of such structured approaches to decision-making about rehabilitation and analysing their effect on clinical practice and outcomes for patients.

## Data Availability

The datasets used and analysed during the current study are available from the corresponding author on reasonable request.

## Abbreviation

PPI: Patient and Public Involvement
